# Loyalty in the time of COVID-19: A review of the literature in tourism destination settings

**DOI:** 10.3389/fpsyg.2023.1119737

**Published:** 2023-02-15

**Authors:** Oliver Cruz-Milán

**Affiliations:** Department of Management and Marketing, College of Business, Texas A&M University-Corpus Christi, Corpus Christi, TX, United States

**Keywords:** COVID-19, loyalty, destination, tourism, literature review, explanation, prediction, theory

## Abstract

Tourism destinations constitute a conglomerate of attractions, service providers, and retailers that make up the overall offerings and experiences that attract visitors. However, given the severe consequences that the COVID-19 pandemic has had on the tourism industry, it is crucial to appraise consumer loyalty towards destinations in the context of the coronavirus disruptions. An increasing number of academic works examining the factors that influence destination loyalty have been carried out since the pandemic breakout, but no evaluation of their cumulative results and findings has been offered in the literature. Therefore, this research conducts a review of studies that have empirically investigated the drivers of destination loyalty during the pandemic in diverse geographical settings. By analyzing 24 journal articles selected from the Web of Science (WoS) database, this work adds to the literature by providing an assessment of the state-of-the-art body of knowledge about the explanation and prediction of loyalty for tourism destinations in the context of COVID-19.

## Introduction

Building consumer loyalty is an essential objective for managers and is among the core relational constructs that have drawn greater attention in marketing and consumer behavior (Keller, 2013; Solomon, 2015). For instance, the importance of loyalty in retailing has been investigated by marketing scholars as a way to understand its drivers and explanatory frameworks (Dunne et al., 2014). In a parallel form in which customer loyalty is developed for retailing businesses and other service providers, tourist loyalty can also be formed towards the places where people travel to. However, unlike conventional goods, services, and/or stores, tourism destinations represent products and experiences concentrated in a given geographical location, with numerous private and public stakeholders, and fragmented marketing activities by many entities (Gursoy et al., 2009; Pike, 2012). Destinations are bundles of complex, dynamic ecosystems in which economic forces, environmental settings, and visitor-resident interactions contribute to shape consumer attitudes and responses (Pike, 2005; Ritchie and Crouch, 2011). At the outset of 2020 the health pandemic caused by COVID-19 (or SARS-CoV-2) generated a worldwide crisis that suddenly disrupted the travel and tourism industry (World Tourism Organization, 2020). The sanitary measures imposed by authorities severely damaged tourism-reliant regions that suffered a drastic drop in visitors due to the suspension/reduction of operations and the fear of infection by travelers (Sigala, 2020; Fotiadis et al., 2021; Gössling et al., 2021). Against this backdrop, the way in which the coronavirus overall threat has affected the degree of consumer loyalty for tourism destinations remains unknown.

Destination loyalty can be defined as the “behavioral consistency of repeated visits to a destination fueled by a psychological desire to visit the destination “(Niininen, 2022, p. 870). Over the years, consumer loyalty has been studied in the travel and tourism academic literature identifying the processes and phases of loyalty building. Based mostly on the works of Jacoby and Chestnut (1978) and Oliver (1999), loyalty in the context of destinations has been conceptualized in terms of attitudinal and behavioral dimensions by Back and Parks (2003), or as a composite combining both dimensions according to Oppermann (2000). Tourist attitudinal loyalty is usually operationalized through cognitive (thoughts/beliefs), affective (feelings/emotions), and conative (intentions/willingness) components, while behavioral loyalty is measured through overt actions (actual visits to the destination). In this respect, given the uniqueness and peculiarity of tourism contexts (Gartner, 2014; Williamson and Hassanli, 2022) and the need to better understand the impact of the pandemic on travel (World Travel and Tourism Council, 2021), the objective of this research is to provide an integrated, updated view of the body of knowledge generated about destination loyalty since COVID-19 appeared.

By conducting a review of empirical results from studies elaborated during the pandemic, this work contributes to identify the drivers of destination loyalty as the focal construct, supporting the advancement of marketing theory based on inductive-statistical explanations (Hunt, 2010). Understanding how different variables are organized in nomological networks to predict consumer behavior permits building theory by means of new hypothesis-testing, replication studies, and the potential to generate empirical generalizations (Colquitt and Zapata-Phelan, 2007; Corley and Gioia, 2011; Calder et al., 2019). This type of works represents a valuable contribution by synthetizing empirical results that extend the boundaries and conditions of extant knowledge, which adds to the development of theoretical frameworks in marketing and management (Whetten, 1989; Ladik and Stewart, 2008) particularly in an unprecedented scenario such as the COVID-19 pandemic (Yang et al., 2021). According to Williamson and Hassanli (2022), various types of destination loyalty are: homogeneous (to a single destination), horizontal (to other similar destinations), vertical (to providers at different levels of the tourism system), and experiential (to a holiday style, activity, or experience independent of a specific location). For the purpose of this work, the focus of the review is on homogeneous loyalty, reflected as the desire to return to the same, previously visited destination.

## Review approach

The review of the literature was conducted through the Web of Science (WoS) platform (JCR, 2022), as in recent marketing and tourism systematic analyses (e.g., Gupta et al., 2022; Liu et al., 2022). The search query of key terms in English (“loyalty” + “destination” + “COVID” + “tourism”) specified the date ranges from November 1, 2019, the month when COVID-19 presumably appeared (Myoung, 2022) to November 1, 2022. The initial search yielded 57 records, of which 3 contained titles and/or abstracts in English, but their content was written in other languages (two in Spanish and one in German). Out of the 57, 33 works were excluded from further analysis because they did not comply with all the following criteria: (a) empirical research with data collected during the specified range period; (b) quantitative operationalization of the loyalty construct; (c) focused on tourism destinations, rather than individual products or business sectors (e.g., hotels, airlines, cruises); (d) framed in the context of the COVID-19 pandemic (e.g., research results and/or implications). The remaining 24 journal articles that were ultimately reviewed are provided in Table 1.

**Table 1:** Published research reviewed.

Authors/Journal	Destination context	Sample size	Sample characteristics	Data collection period	Analysis/software	Loyalty construct	Variance explained
Chebli et al. (2021) *Journal of Tourism and Services*	Algeria (Sahara)	*n* = 123	Domestic tourists	January–February, 2021	CB-SEM (AMOS)	Intention to revisit: 1 item (conative)	R^2^ = N/A
Zaman et al. (2021) *Cogent Business & Management*	South Korea (various destinations)	*n* = 266	Expatriates living in South Korea	January–May, 2020	PLS-SEM (SmartPLS)	Destination loyalty: 3 items (cognitive, affective, conative)	R^2^ = 0.355
Hassan and Soliman (2021) *Journal of Destination Marketing & Management*	Egypt	*n* = 543	Domestic tourists	April–May, 2020	PLS-SEM (WarpPLS)	Revisit intention: 3 items (conative, affective)	R^2^ = 0.690
Garcia-Reinoso et al. (2021) *El Periplo Sustentable*	Ecuador (Manta)	*n* = 484	Domestic tourists	April–May, 2020	Cluster analysis (SPSS)	Intentions to return: (items not shown in article)	R^2^ = N/A
Woosnam et al. (2021) *Journal of Travel & Tourism Marketing*	Last destination visited during the pandemic by participants in USA	*n* = 600	Domestic tourists	June–August, 2020	CB-SEM (EQS)	Destination loyalty: 3 items (conative)	R^2^ = 0.360
Han et al. (2021) *Frontiers in Psychology*	China	*n* = 456	Domestic tourists	December, 2020	CB-SEM (AMOS), mediation (PROCESS)	Tourist loyalty: 3 items (conative)	R^2^ = 0.942
Rather (2021) *Journal of Destination Marketing & Management*	India (Jammu and Kashmir)	*n* = 325	Not specified	June–July, 2020	PLS-SEM (SmartPLS), mediation (PROCESS)	Revisit intention: 3 items (conative, affective)	R^2^ = 0.690
Kralikova et al. (2021) *European Countryside*	Czech Republic (Moravian wine region)	*n* = 345	Domestic tourists	May–June, 2020	OLS multiple regression	Revisit intention: (items not shown in article)	R^2^ = N/A
Suhartanto et al. (2022) *Tourism Recreation Research*	Indonesia	*n* = 300	Domestic tourists	January–February, 2021	PLS-SEM (SmartPLS)	Intention to visit the destination: 2 items (conative)	R^2^ = 0.485
Manchanda and Deb (2022) *Current Issues in Tourism*	Destination visited through virtual reality tourism applications	*n* = 484	Not specified	November–December, 2020	CB-SEM (AMOS)	Intention to physically visit the destination: 3 items (conative)	R^2^ = N/A
Tu et al. (2022) *SAGE Open*	China (Gaochun District)	*n* = 375	Domestic tourists	December, 2020	CB-SEM (AMOS)	Tourist behavioral intentions: 5 items (conative)	R^2^ = N/A
Torabi et al. (2022) *Sustainability*	Iran (Tehran)	*n* = 380	Domestic tourists	May, 2020	PLS-SEM (SmartPLS)	Intention to revisit: 3 items (conative)	R^2^ = 0.373
Koç et al. (2022) *Journal of Destination Marketing & Management*	Turkey (Pamukkale)	*n* = 256	Domestic tourists	August–September, 2020	PLS-SEM	Revisit intention: 5 items (conative)	R^2^ = 0.296
Papadopoulou et al. (2022) *Journal of Travel Research*	Various Mediterranean destinations	*n* = 582	Domestic and international tourists	May, 2020	CB-SEM (Mplus), moderation (PROCESS)	Intention to revisit and recommend: 5 items (conative)	R^2^ = 0.820
Lin et al. (2022) *Current Issues in Tourism*	Destination visited during the pandemic by participants in China	*n* = 283	Domestic tourists	November, 2021	CB-SEM (AMOS)	Destination loyalty: 4 (conative, cognitive)	R^2^ = N/A
Nie et al. (2022) *Journal of Destination Marketing & Management*	China (Nanjing)	*n* = 535	Domestic tourists	April–May, 2021	PLS-SEM (SmartPLS)	Loyalty: 5 items (conative)	R^2^ = 0.323
Otero-Gomez and Giraldo-Perez (2022) *Revista Universidad & Empresa*	Colombia (Villavicencio)	*n* = 130	International tourists	August–September, 2021	Spearman’s rho correlations (JASP)	Revisit intention: 4 items (conative)	R^2^ = N/A
Carvache-Franco et al. (2022a) *Sustainability*	Ecuador (Santa Elena)	*n* = 318	Domestic and international tourists	April–June, 2021	Multiple regression (SPSS)	Return intentions: 1 item (conative)	R^2^ = 0.358
Carvache-Franco et al. (2022b) *Sustainability*	Costa Rica (Jacó)	*n* = 304	Domestic and international tourists	June, 2021	Multiple regression (SPSS)	Return intentions: 1 item (conative)	R^2^ = 0.234
Lee and Kim (2022) *Sustainability*	South Korea	*n* = 774	International students in South Korea	Not specified. The study operationalized constructs related to COVID-19	PLS-SEM (SmartPLS)	Place loyalty: 4 items (conative)	R^2^ = 0.423
Cambra-Fierro et al. (2022) *European Research on Management and Business Economics*	Peru (Lima)	*n* = 250	Not specified	December, 2020-January, 2021	PLS-SEM (SmartPLS)	Destination loyalty: 4 items (conative, affective)	R^2^ = 0.435
Majeed et al. (2022) *Tourism and Hospitality Research*	Destination previously visited by participants in China	*n* = 579	Not specified	October, 2020	EFA and CFA	Destination brand choice / loyalty: 5 items (conative, cognitive)	R^2^ = N/A
Šerić and Mikulić (2022) *Tourism Review*	Croatia	*n* = 333	International tourists	Summer–Fall, 2021	PLS-SEM	Brand loyalty: 4 items (cognitive, affective, conative)	R^2^ = N/A
Huete-Alcocer and Hernandez-Rojas (2022) *Journal of Retailing and Consumer Services*	Spain (Córdoba)	*n* = 154	Not specified	November, 2021	PLS-SEM (SmartPLS)	Loyalty to destination: 4 items (conative)	R^2^ = 0.604

Journal titles are shown in italics. Software used for statistical analysis are in parentheses when reported. EFA = Exploratory factor analysis; CFA = Confirmatory factor analysis; CB-SEM = Covariance-based structural equation modeling; PLS-SEM = Partial least squares structural equation modeling; OLS=Ordinary Least Square; R2 = Coefficient of determination; N/A = Not available.

## Results

### Descriptive analysis

The analysis of articles shown in Table 1 revealed that all studies operationalized destination loyalty as attitudinal loyalty, with coefficients of determination (R^2^) ranging from 0.234 to 0.942. The outcome variables employed scale items corresponding to conative, affective, and/or cognitive loyalty to elicit revisit intentions or likelihood to return to the same destination. The operationalization of destination loyalty typically included scale items referring to recommendation or positive word-of-mouth (WOM) about the destinations, combined with other revisit intentions items within the same loyalty construct. However, some investigations specified and operationalized tourists’ recommendations or endorsements as additional, separate constructs in their models (e.g., Chebli et al., 2021; Kralikova et al., 2021; Koç et al., 2022; Suhartanto et al., 2022; Carvache-Franco et al., 2022a,b).

All 24 articles reported the use of survey-based methods for data collection (electronically and in-person), with samples ranging from 123 to 774 (average sample size = 382). As indicated in Table 1, the majority of the articles reported that their survey participants were domestic tourists, which is not surprising due to travel restrictions and border closures during 2020. Indeed, most studies that used samples of international tourist respondents collected their survey data later in 2021 (e.g., Otero-Gomez and Giraldo-Perez, 2022; Šerić and Mikulić, 2022; Carvache-Franco et al., 2022a,b). Of the reviewed studies, 7 utilized co-variance based structural equation modeling (CB-SEM), while 11 employed partial least squares structural equation modeling (PLS-SEM), in line with the growing popularity of PLS-SEM in tourism marketing research (Hair et al., 2021). The rest of the works reported other analysis techniques (e.g., multiple regression, cluster analysis, correlations). In terms of the geographical contexts, the investigated destinations correspond to countries in the Americas (North, Central, and South America), Europe, Northern Africa, the Middle East, as well as Asia (South, East, and Southeast Asia), reflecting the wide diversity of study settings in which the research projects were developed.

The reviewed studies were published mostly in tourism-oriented journals, some of which are included in the Australian Business Deans Council list (ABDC, 2019) rated “A*” (*Journal of Travel Research*), rated “A” (*Current Issues in Tourism*, *Journal of Destination Marketing & Management*, *Journal of Retailing and Consumer Services*, *Journal of Travel & Tourism Marketing*, *Tourism Recreation Research*), or rated “B” (*Tourism and Hospitality Research*, *Tourism Review*). Three journals published more than one of the works reviewed: *Sustainability* (five articles), *Journal of Destination Marketing & Management* (three articles), and *Current Issues in Tourism* (two articles). The fact that two studies were published in Spanish language journals by Otero-Gomez and Giraldo-Perez (2022) in *Revista Universidad & Empresa*, and by Garcia-Reinoso et al. (2021) in *El Periplo Sustentable* reflects the growing interest of academics based in Latin America to disseminate their research to broader audiences through journals indexed in international databases (Cruz-Milán, 2014). The review identified recurrent theories and conceptual frameworks under which the hypotheses were developed. Examples of some well-known theories employed are Mehrabian and Russell’s (1974) stimulus-organism-response (S-O-R) in Koç et al. (2022), Majeed et al. (2022), and Nie et al. (2022), Rogers’ (1975) protection motivation theory (PMT) in Rather (2021) and Cambra-Fierro et al. (2022), or Ajzen’s (1991) theory of planned behavior (TPB) in Torabi et al. (2022), adding to the integration of research findings with higher-level theoretical explanations (Hunt, 2010).

### Predictors of destination loyalty

**Figure 1:**
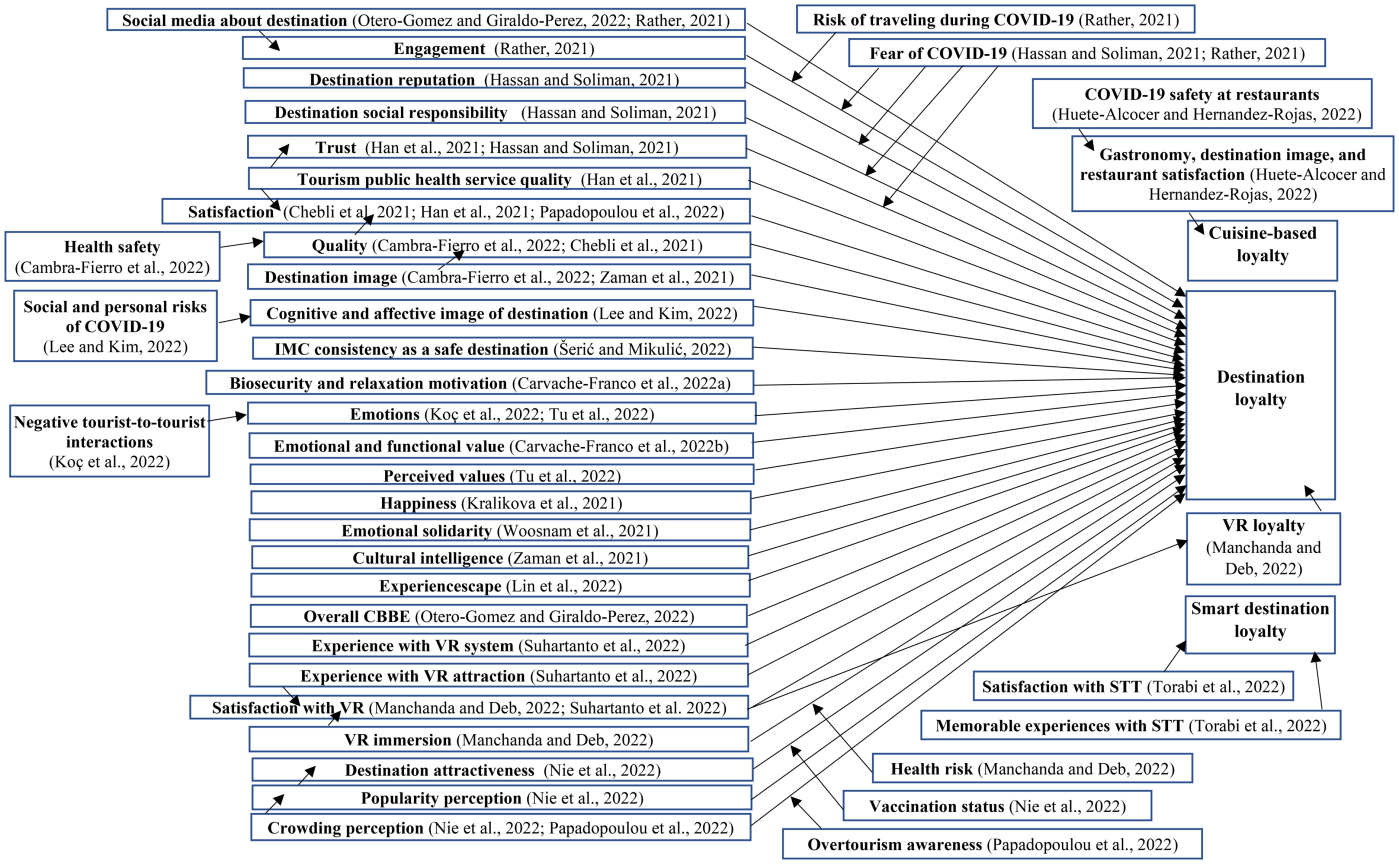
Drivers of destination loyalty.

The review of the research results reported in the articles reveal a variety of constructs that predict destination loyalty as illustrated in Figure 1. The determinants of destination loyalty found as statistically significant with available effects as Beta coefficients (Vogt and Johnson, 2016) were: satisfaction (β = 0.790) by Papadopoulou et al. (2022); destination image (β = 0.530) by Zaman et al. (2021); emotional and functional value (β = 0.477) by Carvache-Franco et al. (2022b); perceived values (β = 0.382) and emotions (β = 0.434) by Tu et al. (2022); happiness (β not reported) by Kralikova et al. (2021); cultural-archaeological and sun-beach motivations (β not reported) by Garcia-Reinoso et al. (2021); emotional solidarity’s dimensions of feeling welcomed (β = 0.560), emotional closeness (β = 0.240), and sympathetic understanding (β = 0.530) by Woosnam et al. (2021); tourists’ cultural intelligence (β = 0.166) by Zaman et al. (2021); destination experiencescape’s dimensions of key attractions (β = 0.250) auxiliary elements (β = 0.380), and atmosphere (β = 0.230) by Lin et al. (2022); and the overall consumer-based brand equity (CBBE) of the destination (β = 0.590) by Otero-Gomez and Giraldo-Perez (2022).

While the previously mentioned constructs are among those usually found in the tourism marketing and destination loyalty literature, other research models examined the effects of constructs more closely operationalized to measure consumers’ perceptions and attitudes specifically related to COVID-19. For instance, Otero-Gomez and Giraldo-Perez (2022) demonstrated the impact of information posted on social media about the destination during the pandemic (β = 0.263) on destination loyalty. The study by Rather (2021) also found the effect of social media information about the destination (β = 0.610) as an antecedent of tourist’s engagement, which in turn had an impact (β = 0.630) on destination loyalty, exhibiting a partial mediation. In Rather’s (2021) work, the impact of engagement on destination loyalty was negatively moderated by risk of traveling during COVID-19 (−0.032) and fear of COVID-19 (−0.037). Research by Hassan and Soliman (2021) examined a model in which loyalty was determined by the destination’s reputation (β = 0.367), social responsibility (β = 0.227), and tourists’ trust (β = 0.293). Hassan and Soliman (2021) also found that tourists’ fear of COVID-19 moderated the effects of social responsibility (0.143), reputation (−0.121) and trust (−0.075) on destination loyalty.

Han et al. (2021) investigated destination loyalty predicted by the degree of tourism public health service quality (β = 0.172), tourists’ trust (β = 0.240), and satisfaction with the destination (β = 0.615). The model showed that the latter two constructs mediated the effects between public health service quality and destination loyalty. Chebli et al. (2021) found tourists’ loyalty was determined by satisfaction (β = 0.688), which in turn was preceded by the perceived quality’s dimensions of physical/scenic environment (β = 0.233), relational environment (β = 0.213), entertainment (β = −0.881) and reliability/governance (β = 0.363) at the destination. Similarly, the research by Cambra-Fierro et al. (2022) concluded that destination loyalty is explained by perceived quality (β = 0.660), which in turn is predicted by the destination’s image (β = 0.543) and perceived health safety (β = 0.194) producing indirect (mediation) effects. Other constructs specifically operationalized for the pandemic context identified in the review were the degree of consistency of integrated marketing communications (IMC) as a safe destination (β = 0.408) examined by Šerić and Mikulić (2022), and the biosecurity (β = 0.185) and relaxation (β = 0.404) motivations used in the model by Carvache-Franco et al. (2022a).

The perceived crowding at destinations was found to negatively impact (β = −0.180) tourist’s destination loyalty in the research of Papadopoulou et al. (2022), and the relationship is moderated by overtourism awareness (β = 0.300). Similarly, the model estimated by Nie et al. (2022) revealed destination loyalty not only influenced by the extent of crowding at the destination (β = −0.144), but also by its perceived popularity (β = 0.172) and attractiveness (β = 0.464). Interestingly, a moderation through multi-group analysis (MGA) comparing COVID-19 vaccination status (vaccinated vs. non-vaccinated) yielded a greater, positive effect of attractiveness on loyalty in tourists who had been vaccinated. The study by Lee and Kim (2022) demonstrated the impact of cognitive place image (β = 0.170) and affective place image (β = 0.535) as immediate antecedents of destination loyalty. In the same model, the authors found cognitive place image predicted by social risks of COVID-19 (β = −0.135) and personal risks of COVID-19 (β = 0.140), while the affective place image was determined by social risks perceptions of COVID-19 (β = −0.119) through the assessment of mediation effects. Similarly, Koç et al. (2022) evidenced the impact of the positive emotions such as joy (β = 0.250) and positive surprise (β = 0.201) on destination loyalty, and demonstrated that the influence of negative tourist-to-tourist interaction at the destination on loyalty is mediated by joy (−0.076).

Three of the reviewed works addressed technological innovations used by consumers and also as part of the destination’s offerings. Suhartanto et al. (2022) showed the influence that virtual reality (VR) has on intentions to physically “return” to the destination as determined by experience with VR system (β = 0.186), experience with VR attraction (β = 0.178), and satisfaction with VR (β = 0.401). Manchanda and Deb (2022) also researched the use of multisensory VR technology and found destination loyalty predicted by VR immersion (β = 0.479) and satisfaction with VR (β = 0.096), with the statistically significant moderation of health risk (−0.477) between VR immersion and loyalty. Further, Manchanda and Deb (2022) found partial mediation effects of satisfaction with VR between VR immersion and destination loyalty, and of VR loyalty between satisfaction with VR and destination loyalty. Torabi et al. (2022) operationalized loyalty towards destinations with smart tourism technologies (STTs), which was determined by memorable experiences with STT (β = 0.421) and satisfaction with STT (β = 0.243). Another special case of loyalty operationalization was in the research by Huete-Alcocer and Hernandez-Rojas (2022), who found cuisine-based destination loyalty formed by the overall image of the destination (β = 0.219), its local gastronomy (β = 0.251), and satisfaction with restaurants (β = 0.403), all of which are in turn predicted by COVID-19 safety measures at restaurants. Finally, Majeed et al. (2022) developed the destination brand image and tourist behavior (DBITB) scale which included a dimension corresponding to destination choice/loyalty, but no prediction of external constructs was reported.

## Conclusion

Due to the lack of a synthesis about research on tourism destination loyalty in COVID-19 settings, a literature review was conducted by examining 24 journal articles published in 2021 and 2022. Although the search for studies encompassed the time period since the coronavirus appeared, none of the 24 studies was published with an assigned volume/issue during 2020. It seems logical that when the pandemic crisis first broke out early in 2020 (first epidemic wave), many authors devoted their work to design, execute, write, and submit their research for peer-review, which ultimately led to final journal publication in the following years. In this respect, it should be noted that the effects of the constructs in the estimated loyalty models could have been influenced by the distinct time periods in which survey-data was obtained for each study. The perceived threat of infection according to fluctuations in coronavirus waves (upward or downward trends) has an impact on tourists’ risk assessments and intentions to travel (Fotiadis et al., 2021). Considering the lack of consensus on the criteria for determining the duration of epidemic waves (Zhang et al., 2021) which manifest heterogeneously across countries depending on COVID-19 variants (Dhama et al., 2023) and other factors (e.g., geography, population, institutional measures, vaccination rates), it is uncertain the extent to which destination loyalty was affected by the timing in which data was collected in each country.

While some of the articles studied destination loyalty drawing from existing models in the marketing and tourism literature framed within the context of COVID-19 impacts, others works hypothesized moderation and/or mediation effects with new constructs specifically relevant to the pandemic disruptions (e.g., Han et al., 2021; Hassan and Soliman, 2021; Rather, 2021; Cambra-Fierro et al., 2022; Lee and Kim, 2022; Manchanda and Deb, 2022; Nie et al., 2022; Papadopoulou et al., 2022). The overall results from the reviewed models show the substantial impact that some drivers continue to have on loyalty, as exhibited by the effects from destination satisfaction (β = 0.790) by Papadopoulou et al. (2022) and (β = 0.688) by Chebli et al. (2021), perceived quality (β = 0.660) by Cambra-Fierro et al. (2022), engagement (β = 0.630) by Rather (2021), or destination image (β = 0.530) by Zaman et al. (2021). This serves as corroboration about key constructs that meta-analytic studies prior to the pandemic identified as determinant on destination loyalty, such as satisfaction (Ladeira et al., 2016) or destination image (Zhang et al., 2014). Further, despite the various effects induced by the novel coronavirus-related constructs were generally smaller (e.g., perceived health safety, destination crowding, risks and fear of COVID-19, vaccination status), such findings add to the literature by providing evidence about their role in swaying tourists’ loyalty in pandemic contexts. This is because the incorporation of such variables in mediation and conditional process models allows to enhance the prediction of the focal outcomes (Woodside, 2017), and in this case contributes to the better explanation of loyalty and thus a greater understanding of phenomena and theory building (Kumar et al., 2013).

Nonetheless, it appears that loyalty towards destinations in some parts of the world could have been additionally influenced by the restrictions imposed on overseas travel to certain regions during the pandemic. This is suggested by the large variance explained in the loyalty outcomes reported for highly populated countries with significant domestic markets such as China (Han et al., 2021), India (Rather, 2021), or some in the European area (Papadopoulou et al., 2022) that are typically strong outbound tourism countries. For instance, the border closures in some countries produced an inflow of foreign travelers to other destinations with little restrictive health controls, as was the case of the Mexican Caribbean region in which returning visitors became a decisive factor to keep economic and business activity during the pandemic (Cruz-Milán and Lagunas-Puls, 2021). In this respect, sanitary-related policies established by authorities along with travelers’ cautionary measures seem to give way to emerging models of behavior conditioned by pandemic threats, requiring research programs to better understand how tourist segments and their loyalty towards destination may be altered (Zenker and Kock, 2020; Miao et al., 2021).

### Limitations and further research

The literature review was performed based on the records provided by the Web of Science (WoS) database derived from key term queries in English. Therefore, studies that could have been obtained by searching key terms in other languages were not included in the review. Further, the 24 works analyzed correspond to studies published in the form of journal articles, so other types of research in books, chapters, conference proceedings, or dissertations/theses were out of the scope of the review. The findings of the studies were based on convenience samples, which calls for caution in inferring generalizations given the limitations of non-probabilistic sampling (Vogt and Johnson, 2016). In terms of potential research avenues, the reviewed models estimated the effects of some constructs typically specified in the literature (e.g., satisfaction, trust, values), but other constructs such as commitment or involvement in the loyalty explanation chain could be incorporated in future studies (Vásquez-Párraga and Sahagún, 2020). Additionally, since none of the investigations employed measures of behavioral loyalty variables in the analyses, further research is necessary using actual visitation through self-reported measures (e.g., post-trip surveys), secondary data (e.g., tourist arrival records) or big data analytics (e.g., GPS-mobility).

The review of journal articles found that only one of the examined models specified cognitive and affective dimensions as drivers of destination loyalty (Lee and Kim, 2022). Hence, future studies may assess the impact of health and safety risks on consumer loyalty encompassing other constructs along the cognitive-affective routes in the formation of CBBE (Keller, 2016) in the context of tourism destinations (Tasci, 2021). Similarly, this review identified that one of the works investigated destination loyalty in terms of the value provided by the gastronomic and restaurant offerings (Huete-Alcocer and Hernandez-Rojas, 2022), which calls for further research exploring the specific role of other businesses and retailers (e.g., hotels, entertainment venues, shopping centers, theme parks) in building tourism destination loyalty. It is also recommended to implement longitudinal research designs, or studies through experimental/quasi-experimental approaches (Stoner et al., 2022) as a way to better ascertain cause-effect relationships (Kerlinger and Lee, 2000; Hunt, 2010).

## Author contributions

OC-M conceived, designed the concept, collected the data, wrote the manuscript, and read and agreed to the published version of the manuscript.

## Funding

This publication was supported by the TAMU-CC Open Access Publication Fund.

## Conflict of interest

The author declares that the research was conducted in the absence of any commercial or financial relationships that could be construed as a potential conflict of interest.

## Publisher’s note

All claims expressed in this article are solely those of the authors and do not necessarily represent those of their affiliated organizations, or those of the publisher, the editors and the reviewers. Any product that may be evaluated in this article, or claim that may be made by its manufacturer, is not guaranteed or endorsed by the publisher.
